# Model integrating CT-based radiomics and genomics for survival prediction in esophageal cancer patients receiving definitive chemoradiotherapy

**DOI:** 10.1186/s40364-023-00480-x

**Published:** 2023-04-24

**Authors:** Jinfeng Cui, Li Li, Ning Liu, Wenhong Hou, Yinjun Dong, Fengchang Yang, Shouhui Zhu, Jun Li, Shuanghu Yuan

**Affiliations:** 1 Center for Medical Integration and Practice, Shandong University, Jinan, Shandong China; 2 Department of Radiation Oncology, Shandong Cancer Hospital and Institute, Shandong First Medical University and Shandong Academy of Medical Sciences, Shandong Cancer Hospital Affiliated to Shandong First Medical University, Jinan, Shandong China; 3 Department of Thoracic Surgery, Provincial Hospital Affiliated to Shandong First Medical University, Jinan, Shandong China; 4 Department of Radiation Oncology, The Affiliated Cancer Hospital of Zhengzhou University, Zhengzhou, Henan China; 5 Department of Radiation Oncology, Shandong Cancer Hospital Affiliated to Shandong University, Jinan, Shandong China

**Keywords:** Esophageal squamous cell carcinoma, Radiomics, Genomics, Contrast-enhanced computed tomography, Prognosis

## Abstract

**Background:**

Definitive chemoradiotherapy (dCRT) is a standard treatment option for locally advanced stage inoperable esophageal squamous cell carcinoma (ESCC). Evaluating clinical outcome prior to dCRT remains challenging. This study aimed to investigate the predictive power of computed tomography (CT)-based radiomics combined with genomics for the treatment efficacy of dCRT in ESCC patients.

**Methods:**

This retrospective study included 118 ESCC patients who received dCRT. These patients were randomly divided into training (n = 82) and validation (n = 36) groups. Radiomic features were derived from the region of the primary tumor on CT images. Least absolute shrinkage and selection operator (LASSO) regression was conducted to select optimal radiomic features, and Rad-score was calculated to predict progression-free survival (PFS) in training group. Genomic DNA was extracted from formalin-fixed and paraffin-embedded pre-treatment biopsy tissue. Univariate and multivariate Cox analyses were undertaken to identify predictors of survival for model development. The area under the receiver operating characteristic curve (AUC) and C-index were used to evaluate the predictive performance and discriminatory ability of the prediction models, respectively.

**Results:**

The Rad-score was constructed from six radiomic features to predict PFS. Multivariate analysis demonstrated that the Rad-score and homologous recombination repair (HRR) pathway alterations were independent prognostic factors correlating with PFS. The C-index for the integrated model combining radiomics and genomics was better than that of the radiomics or genomics models in the training group (0.616 vs. 0.587 or 0.557) and the validation group (0.649 vs. 0.625 or 0.586).

**Conclusion:**

The Rad-score and HRR pathway alterations could predict PFS after dCRT for patients with ESCC, with the combined radiomics and genomics model demonstrating the best predictive efficacy.

## Introduction

The high incidence and mortality of esophageal carcinoma (EC) make this cancer type a major contributor to the global cancer burden [1]. Esophageal squamous cell carcinoma (ESCC) accounts for 90% of patients with esophageal cancer in China, which differs from the predominant occurrence of adenocarcinoma among esophageal cancer patients in Western countries.

Currently, definitive chemoradiotherapy (dCRT) has become the standard of care for inoperable locally advanced ESCC. However, despite rapid advances in radiotherapy techniques, the prognosis of inoperable ESCC remains disappointing. More than 50% of ESCC patients eventually experience disease progression after dCRT, with a 3-year progression-free survival (PFS) rate of only 25–33% [2]. Notably, differences in PFS exist among patients treated with the same dCRT regimen. Thus, the ability to predict PFS after dCRT could help physicians provide individualized treatment for ESCC patients with different risk levels, applying appropriately tailored treatment strategies early for patients at high risk of progression.

In addition to clinical features, radiomics features extracted from pre-treatment contrast-enhanced computed tomography (CT) and gene mutation information extracted from pre-treatment tissues may provide valuable data for predictive models. Radiomics is a non-invasive tool that provides useful additional information by extracting high-throughput quantitative features from images (e.g., CT, magnetic resonance [MR], and positron emission tomography [PET]), which holds great promise for cancer prognosis, diagnosis, and prediction of response to therapy [3–5]. Gong et al. [6] found that a model combining contrast-enhanced CT-based radiomic features and clinical characteristics could predict the recurrence risk of EC among patients treated with dCRT. Larue et al. [7] extracted five radiomic features from CT images before chemoradiotherapy to characterize tumor heterogeneity and found that together the five features could predict the 3-year survival rate of EC patients after neo-chemoradiotherapy. However, these previous studies built predictive models based on clinical risk factors and radiomics features only.

A key feature of cancer cells is a highly unstable genome, which results in the accumulation of somatic mutations in critical oncogenic/oncosuppressor genes that drive uncontrolled cancer cell proliferation [8]. Genetic information has been widely used in the clinic as prognostic biomarkers to predict survival or treatment response in support of clinical decision making [9]. For example, Kirienko et al. [10] demonstrated that radiomics features and gene expression profiles can predict recurrence of non-small cell lung cancer. However, to our knowledge, the potential prognostic value of radiomics features combined with a genetic signature in ESCC patients treated with dCRT has not previously been investigated.

In the present study, using data from pre-treatment contrast-enhanced CT images and genomic analysis of locally advanced stage unresectable ESCC, we attempted to develop and validate radiomics and genomics models for predicting PFS after dCRT in these patients and investigated the value of these models for individual PFS estimation.

## Methods and materials

### Patients

**Fig. 1:**
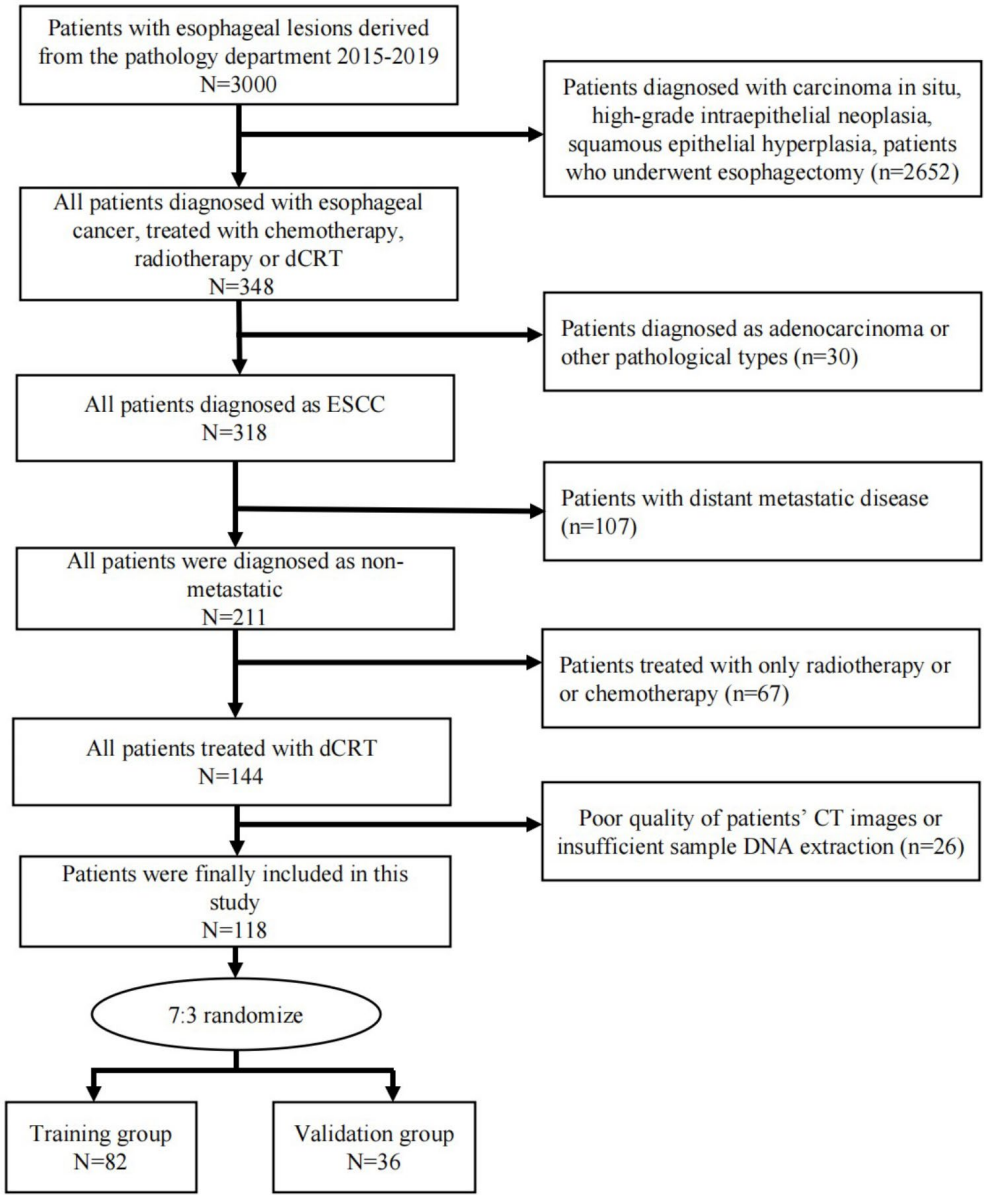
Recruitment and selection process of patients

**Fig. 2:**
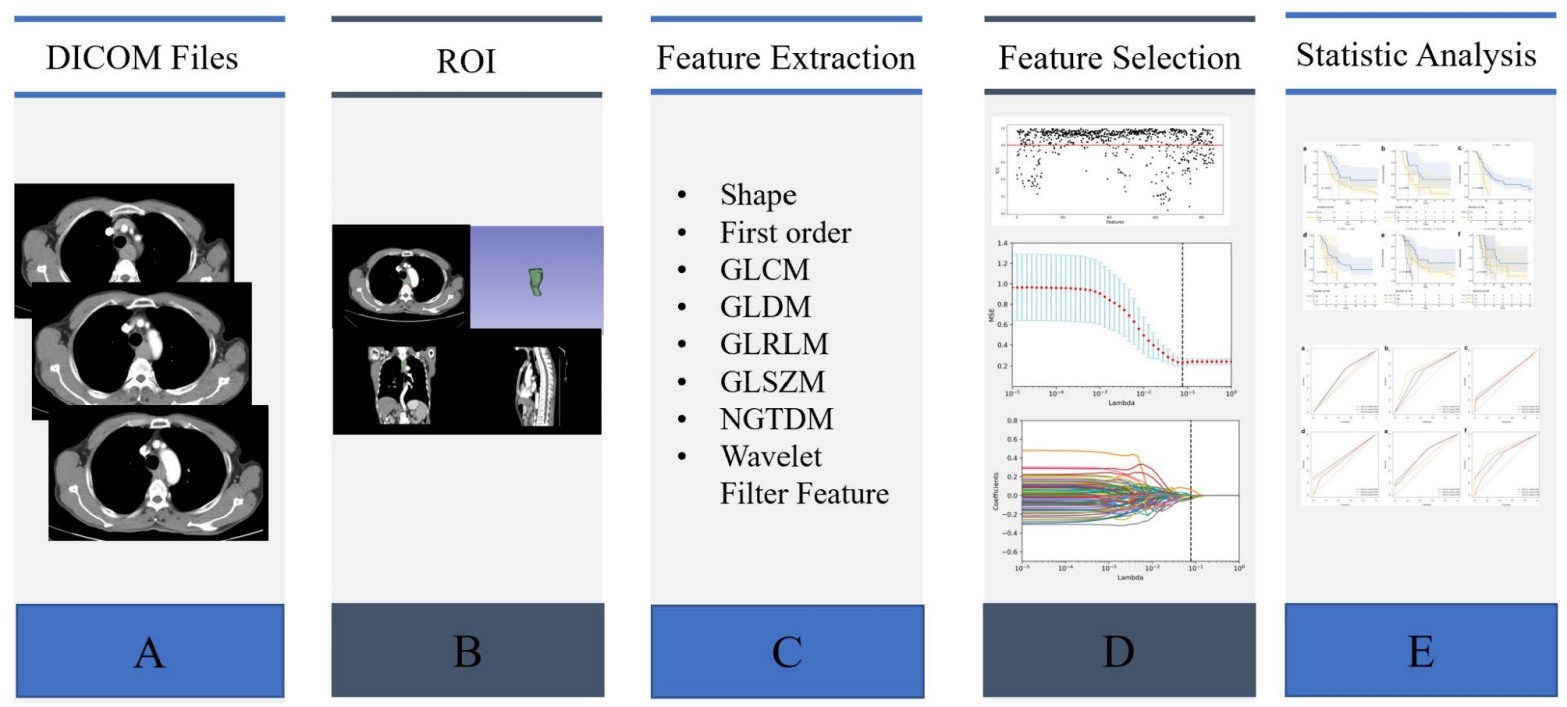
Workflow of the current study

This retrospective study included patients with stage II–III ESCC who received dCRT in our hospital from 2015 to 2019. The inclusion criteria were as follows: (1) pathological diagnosis of ESCC; (2) availability of clinical information, such as alcohol consumption history, smoking status, treatment information, tumor location, survival, etc.; (3) availability of pretreatment contrast-enhanced CT and tumor samples; and (4) treatment with dCRT. The patients recruitment and selection process was showed in Fig. 1. Ultimately, a total of 118 patients were randomized to two groups in a 7:3 ratio, with 82 patients in the training group and 36 patients in the validation group (Table 1). PFS was defined from the date of the pathologic diagnosis of ESCC to the date of progressive disease or death. The workflow of this study is depicted in Fig. 2.

**Table 1:** Comparison of patients’ characteristics between training and validation groups

Variables		Training group (N = 82)	Validation group (N = 36)	p
Gender	Male	59	28	0.508
	Female	23	8	
Age	≤ 65	34	18	0.390
	>65	48	18	
KPS	≤ 80	44	15	0.230
	>80	38	21	
Smoking status	Never	41	18	1
	Former/current	41	18	
Alcohol consumption	Never	52	22	0.812
	Former/current	30	14	
Tumor location	Cervical	4	5	0.203
	Upper thoracic	36	11	
	Middle thoracic	27	15	
	Lower thoracic	15	5	
Clinical stage	II	11	7	0.402
	III	71	29	
Radiation dose	> 60 Gy	13	3	0.546
	> 50.4 Gy ≤ 60 Gy	52	25	
	≤ 50.4 Gy	17	8	
Chemoradiotherapy	SCRT	39	21	0.281
	CCRT	43	15	
Radiation therapy	3D-CRT	14	7	0.756
	IMRT	68	29	
CHEK2	Wild	77	34	1
	Mutation	5	2	
NOTCH2	Wild	75	33	1
	Mutation	7	3	
HRR pathway status	Wild	70	28	0.312
	Mutation	12	8	
Rad-score	≤ 0.36	32	14	0.989
	> 0.36	50	22	

Abbreviations: KPS Karnofsky performance status, SCRT sequential chemoradiotherapy, CCRT concurrent chemoradiotherapy; IMRT intensity modulated radiation therapy, 3D-CRT 3-dimensional conformal radiation therapy

The study was approved by the ethical review committee of our hospital. A completed informed consent form was acquired from each patient in accordance with the guidelines of the Institutional Review Board and the Declaration of Helsinki.

### CT image acquisition

All patients underwent contrast-enhanced CT examination before treatment. A 64-layer spiral CT scanner (Definition AS+, Siemens SOMATOM) was used for CT image acquisition. The scanning parameters were: slice thickness of 5.0 mm, tube voltage of 120 kV, and tube current of 220 mA. Iodinated contrast agent (300 mg/mL) at a dose of 1.5 ml/kg body mass was injected rapidly at a flow rate of 2 mL/s through the patient’s elbow vein using a high-pressure syringe. To ensure more standardized images, we uniformly take patient arterial phase images after contract injection. Enhanced CT images in DICOM format were extracted from the PACS system and used for feature extraction.

### ROI segmentation and radiomics feature extraction

Two radiologists with 10 years of clinical diagnostic experience delineated separately the primary tumor as the region of interest (ROI) on CT images (Fig. 2) using 3D-Slicer software (Version 4.11.0). Primary tumors, defined as lesions with esophageal wall thickening > 5 mm or lumen occlusion diameter > 10 mm and excluding intraluminal gas and oral contrast agent. The two radiologists were blinded to the pathological and clinical data. A total of 851 features were extracted from the manually segmented tumors of each patient using the open-source package Pyradiomics in 3D-Slicer.

### Radiomics feature selection and rad-score construction

The characteristics of each patient were normalized using Z-score normalization to ensure comparability among the data. To minimize any type of bias or overfitting due to an excessive number of features, feature selection was performed in two steps using the inter-class correlation coefficient (ICC) and least absolute shrinkage and selection operator (LASSO) regression. Radiomics features of 20 patients extracted by the two physicians separately were used to calculate ICC. Only the features with ICC ≥ 0.800 were selected for further analysis. The optimum parameter lambda (λ) was selected from the LASSO model using 10-fold cross validation with the minimum mean square error (MSE). The most predictive features and weight coefficients for PFS were selected, and the selected features and corresponding weight coefficients were linearly combined to establish the Rad-score.

### Gene mutation signatures

As our previously described [11], target sequencing of 474 cancer-related genes was performed on tumor tissue samples from each patient. Univariate and multivariate analyses were performed to identify the mutations associated with reduced PFS in ESCC patients. Our result demonstrated that CHEK2 mutations, NOTCH2 mutations, and mutated homologous recombinant repair (HRR) pathways were related to shorter PFS in ESCC patients in univariate analyses, and HRR pathway alterations was independent prognostic factors of PFS in multivariate analyses.

### Construction and validation of models for survival prediction

The receiver operating characteristic (ROC) curve for the ability of the Rad-score to predict PFS was plotted, and the point on the curve with the largest Youden index was selected as the cut-off value for the Rad-score. The optimum selected radiomics features, gene mutation characteristics, and clinical factors were used to construct survival prediction models through Cox proportional hazard regression in the training group. The performance of the models in the training and validation groups was evaluated using the ROC curve and C-index.

### Statistical analysis

The clinical features of patients in the two groups were compared using the chi-squared test. All statistical analyses were conducted using Python version 3.9, R version 4.2.1 and SPSS version 25.0. The Kaplan–Meier method was used to plot PFS curves, and the log-rank test was applied to compare survival differences between the groups. Cox proportional risk regression models were used for univariate and multivariate analyses. The “timeROC” package was applied to draw ROC curves. The “rms” package was applied to calculate the C-index of each prediction model. LASSO regression was implemented using “Python” software. All statistical tests in this study were two-tailed, and p < 0.05 was considered to be statistically significant.

## Results

### Patient characteristics

A total of 118 patients with ESCC who underwent dCRT in our hospital were included in this study. The characteristics of patients in the training and validation groups are presented in Table 1. No significant differences in characteristics were observed between the two groups. After a median follow-up time of 33.4 months, 76 individuals experienced progression. The median PFS in the whole cohort was 11.8 months.

### Rad‑score construction from six radiomics features

A total of 851 features from each patient ROI were extracted using the open-source package Pyradiomics in 3D-Slicer software. The extracted radiomics features included shape features, first order statistical features, gray level co-occurrence matrix (GLCM), gray level dependence matrix (GLDM), gray level run-length matrix (GLRLM), gray level size zone matrix (GLSZM), neighbor gray tone difference matrix (NGTDM), and features obtained via wavelet filtering processing. The mathematical meanings of these radiomic signatures have been described previously [12] and are available at https://pyradiomics.readthedocs.io/en/latest/.

**Fig. 3:**
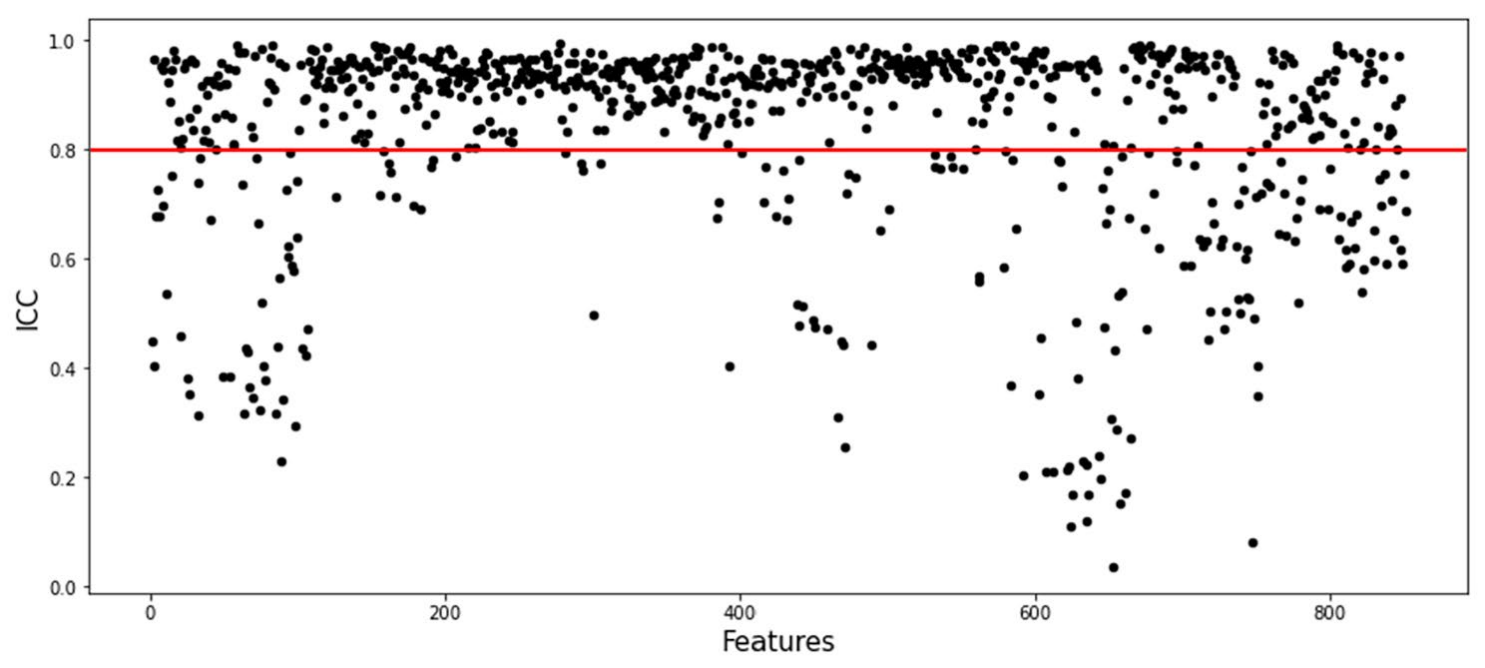
Evaluation of the stability of radiomics features based on ICC. Features with ICC < 0.8 were removed, and the remaining 638 radiomics features were included in the data analysis as stable feature parameters

**Fig. 4:**
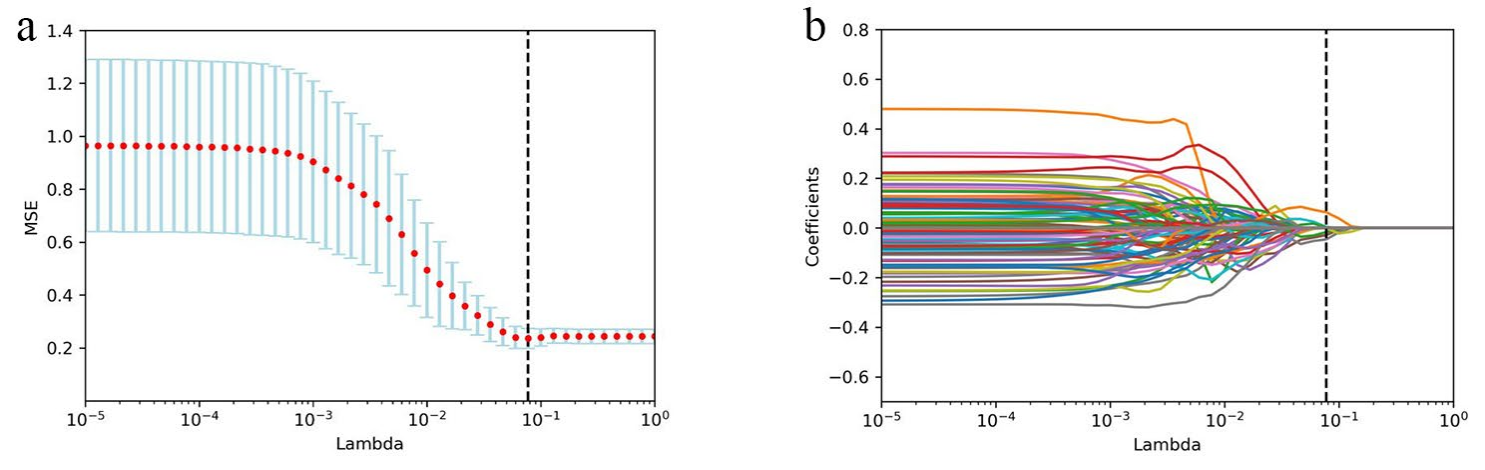
Selection of radiomic features associated with PFS based on LASSO regression models. (**a**): The crossvalidation curve. The vertical axis is mean square error, and the horizontal axis is lambda (λ). (**b**): Coefficient curves for radiomic features. The vertical axis represents the radiomic features’ coefficients, and the horizontal axis is λ

For Rad-score construction, first the features with an ICC < 0.8 were removed (Fig. 3). After this step, 638 radiomics features were included in the subsequent data analysis as stable feature parameters. Next, LASSO regression was applied and identified six radiomics features with non-zero coefficients (Fig. 4a **and b**). These features and the corresponding coefficients are presented in the Table 2. The calculation method for Rad-score based on these features is as follows:

**Table 2:** Radiomics features associated with PFS selected by LASSO regression

Radiomics features	Coefficients
original_shape_SurfaceVolumeRatio	-0.24799156283072044
original_glcm_SumEntropy	-0.1361746133085625
wavelet-LLH_gldm_LargeDependenceLowGrayLevelEmphasis	0.006448868786596698
wavelet-LHL_glcm_Idmn	0.49200413399592363
wavelet-HHL_glszm_GrayLevelVariance	0.1013747131127634
wavelet-LLL_glcm_Imc2	-0.23295948882404996

$$\eqalign{&{\rm{Rad - score}} = \cr & - 0.24799156283072044*{\rm{original\_shape}}\cr&{\rm{\_SurfaceVolumeRatio}}\cr& + - 0.1361746133085625*{\rm{original\_glcm\_SumEntropy}}\cr& + 0.006448868786596698*{\rm{wavelet\_LLH\_gldm}}\cr& {\rm{\_LargeDependenceLowGrayLevelEmphasis}} \cr& + 0.49200413399592363*{\rm{wavelet - LHL\_glcm\_Idmn}}\cr& + 0.1013747131127634*{\rm{wavelet - HHL\_glszm}}\cr& {\rm{\_GrayLevelVariance}}\cr& + - 0.23295948882404996*{\rm{wavelet - LLL\_glcm\_Imc2}}}$$

### Prediction models based on radiomics and genomics features

For the radiomics model for PFS prediction based on the Rad-score, ROC curve analysis identified 0.36 as the optimum cut-off value for the Rad-score. Accordingly, patients with a Rad-score > 0.36 were classified as having a high risk of progression, and those with a Rad-score ≤ 0.36 were classified as having a lower risk of progression. Univariate and multivariate Cox regression analyses then confirmed that the Rad-score was an independent predictor of PFS in the training group (Table 3).

**Table 3:** PFS-related univariate and multivariate analysis in the training group

Variables	Training cohort			
	Univariate analysis		Multivariate analysis	
	HR (95% CI)	p	HR (95% CI)	p
Gender (Male vs. Female)	0.685 (0.364–1.29)	0.241		
Age (≤ 65 vs. >65)	0.853 (0.483–1.509)	0.586		
KPS (≤ 80 vs. >80)	0.871 (0.501–1.513)	0.623		
Smoking status (Never vs. Former/current)	1.231 (0.711–2.132)	0.458		
Alcohol consumption (Never vs. Former/current)	1.674 (0.959–2.922)	0.070		
Tumor location (Cervical reference)		0.294		
Upper thoracic	2.94 (0.396–21.828)	0.292		
Middle thoracic	3.321 (0.443–24.883)	0.243		
Lower thoracic	5.333 (0.67-42.442)	0.114		
Clinical stage (II vs. III)	2.497 (0.988–6.307)	0.053		
Radiation dose (>60 Gy reference)		0.246		
>50.4 Gy ≤ 60 Gy	0.768 (0.375–1.575)	0.471		
≤50.4 Gy	1.374 (0.58–3.253)	0.470		
Chemoradiotherapy (SCRT vs. CCRT)	0.596 (0.341–1.041)	0.069		
Radiation therapy (3D-CRT vs. IMRT)	1.008 (0.499–2.035)	0.983		
CHEK2 (Wild vs. Mutation)	2.658 (1.029–6.867)	0.044		
NOTCH2 (Wild vs. Mutation)	1.77 (0.694–4.515)	0.232		
HRR (Wild vs. Mutation)	2.671 (1.281–5.569)	0.009	2.747 (1.313–5.748)	0.007
Rad-score (≤ 0.36 vs. > 0.36)	2.016 (1.09–3.728)	0.025	2.052 (1.109–3.798)	0.022

Abbreviations: KPS Karnofsky performance status, SCRT sequential chemoradiotherapy, CCRT concurrent chemoradiotherapy; IMRT intensity modulated radiation therapy, 3D-CRT 3-dimensional conformal radiation therapy

For the genomic model for PFS prediction, a gene mutation signature was identified by analysis of the sequencing results for tumor samples from ESCC patients. Univariate analysis identified CHEK2 mutations and mutations in the HRR pathway as related to shorter PFS in ESCC patients in the training group, and the HRR pathway alterations remained independent prognostic factors for PFS on multivariate analyses (Table 4).

Three predictive models for predicting the efficacy of dCRT based on PFS were constructed: the radiomics model, the genomics model, and a model integrating both radiomics and genomic features.

**Table 4:** Predictive performance of the three models in the training group for PFS.

Model	AUC (95%CI)			C-index (95%CI)
	1-year PFS	2-years PFS	3-years PFS	
Rad-score	0.604 (0.490–0.718)	0.605 (0.437–0.774)	0.528 (0.328–0.728)	0.587 (0.516–0.658)
HRR pathway status	0.615 (0.537–0.693)	0.593 (0.540–0.646)	0.589 (0.538–0.640)	0.557 (0.506–0.609)
Rad_HRR	0.676 (0.568–0.783)	0.662 (0.511–0.813)	0.594 (0.415–0.772)	0.616 (0.542–0.690)

Abbreviations: PFS progression-free survival, AUC area under curve

### Predictive performance of the radiomics, genomics, and integrated models

**Fig. 5:**
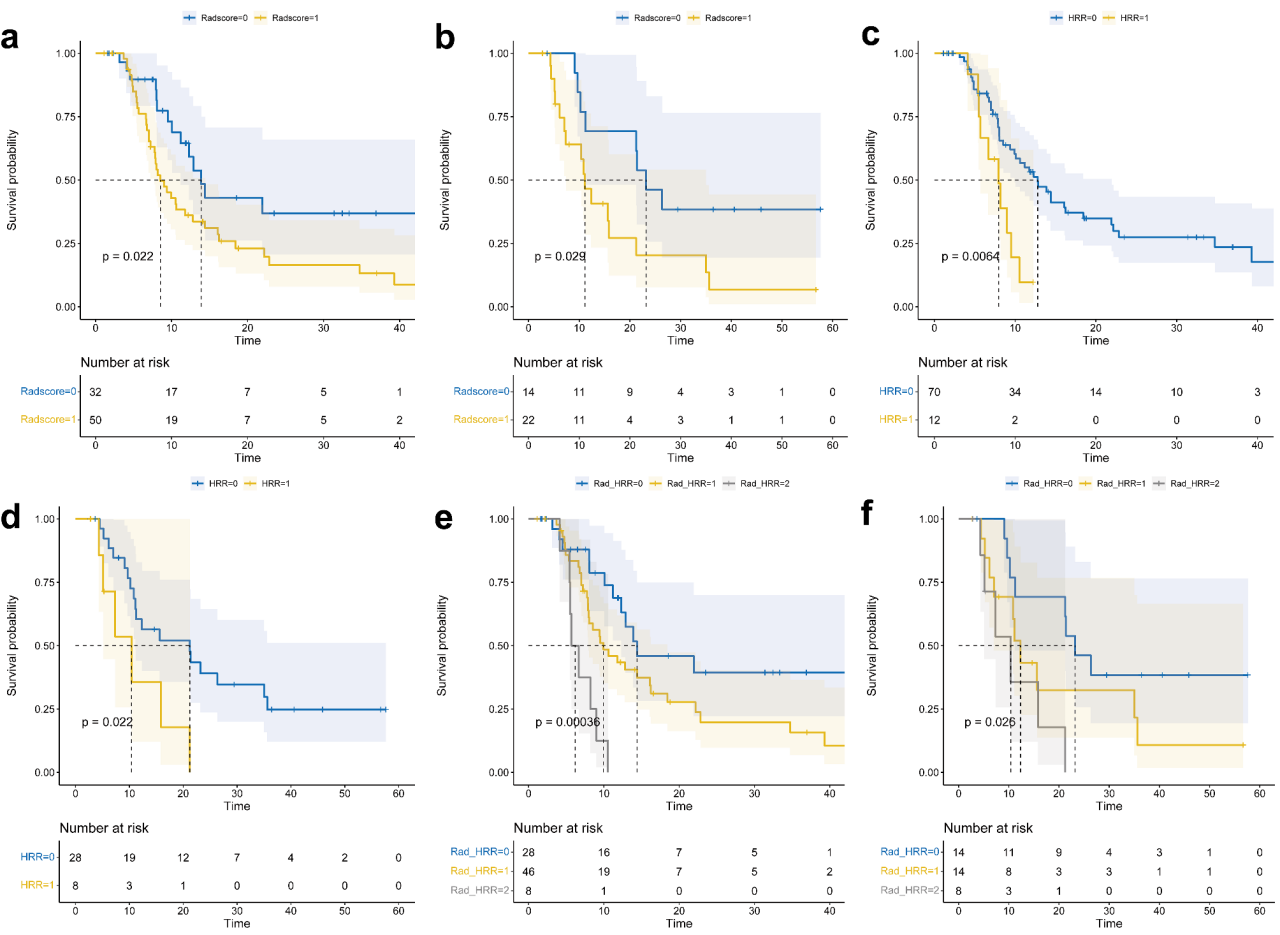
**Kaplan–Meier survival curves constructed based on the three models** Applying the radiomics model, PFS curves for patients with Rad-score ≥ 0.36 (Rad-score = 1) and Rad-score < 0.36 (Rad-score = 0) in the training (**a**) and validation (**b**) groups. Applying the genomics model, PFS curves for patients with HRR pathway mutations (HRR = 1) and without (wild-type, HRR = 0) in the training (**c**) and validation (**d**) groups. Applying the integrated model, PFS curves for patients with high (Rad_HRR = 2), intermediate (Rad_HRR = 1), and low (Rad_HRR = 0) progression risk in the training (**e**) and validation (**f**) groups

**Fig. 6:**
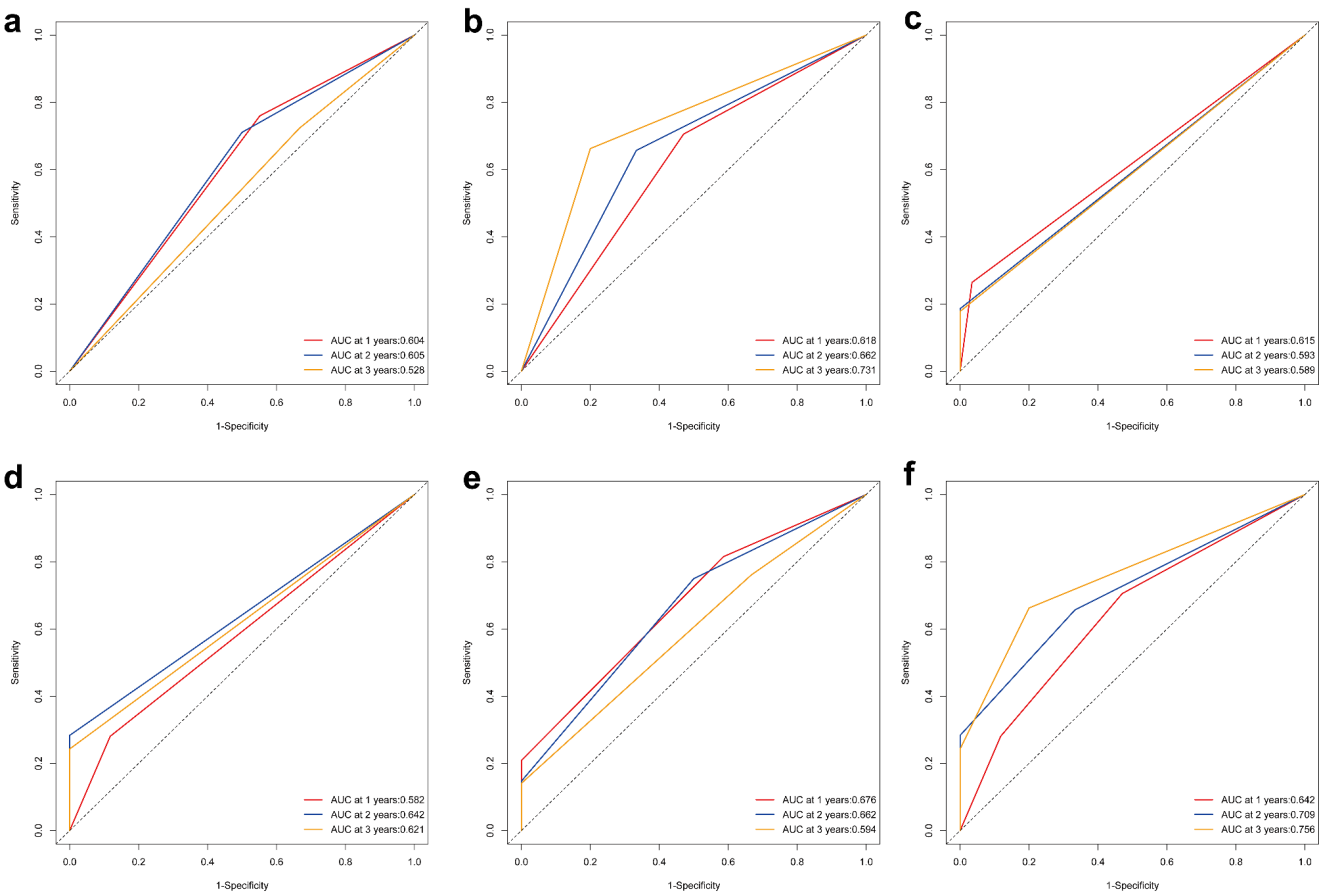
**Evaluation of the predictive performances for the three models for PFS in ESCC patients after dCRT.** Receiver operating characteristic curves showing the predictive performances of the radiomics model (**a, b**), genomics model (**c, d**), and integrated model (**e, f**) in the training and validation groups, respectively

Based on radiomics model, patients in the training with a high risk of progression according to the Rad-score had a significantly shorter median PFS than those with a low risk of progression (Figs. 5a 8.5 months vs. 13.9 months, p = 0.022). The same result was observed in the validation group (Figs. 5b 11.1 months vs. 23.2 months, p = 0.029). The ROC curve analysis results for the performance of the radiomics model for predicting 1-year, 2-year, and 3-year PFS probability are shown in Fig. 6a **and b**. In the training group, the area under the curve (AUC) values for the prediction of 1-year, 2-year, and 3-year PFS probability with this model were 0.604 (95% confidence interval [CI] 0.490–0.718), 0.605 (95% CI 0.437–0.774), and 0.528 (95% CI 0.328–0.728), respectively. In the validation group, the corresponding AUC values were 0.618 (95% CI 0.446–0.789), 0.662 (95% CI 0.474–0.849), and 0.731 (95% CI 0.529–0.933), respectively. The C-index for the radiomics model was 0.587 (95% CI 0.516–0.658) in the training group and 0.625 (95% CI 0.535–0.715) in the validation group.

According to the genomics model, patients with HRR pathway mutations had a significantly shorter median PFS than those without these HRR pathway mutations (wild-type) in the training group (Figs. 5c 7.97 months vs. 12.8 months, p = 0.0064). The same result was observed in the validation group (Figs. 5d 10.4 months vs. 21.2 months, p = 0.022). The ROC curve analysis results for the performance of the genomics model for predicting 1-year, 2-year, and 3-years PFS probability are shown in Fig. 6c **and d**. In the training group, the AUC values for the prediction of 1-year, 2-year, and 3-year PFS probability with this model were 0.615 (95% CI 0.537–0.693), 0.593 (95% CI 0.540–0.646), and 0.589 (95% CI 0.538–0.640), respectively. In the validation group, the corresponding AUC values were 0.582 (95% CI 0.440–0.723), 0.642 (95% CI 0.544–0.740), and 0.621 (95% CI 0.535–0.708), respectively. The C-index for the genomics model was 0.557 (95% CI 0.506–0.609) in the training group and 0.586 (95% CI 0.501–0.671) in the validation group.

In the integrated model combining radiomics features and the genomic signature, patients were separated into high, intermediate, and low progression risk groups based on the Rad-score and HRR pathway mutation status. Patients with a high risk of progression had a significantly shorter median PFS than those with intermediate or low risk of progression in the training group (Figs. 5e and 6.18 months vs. 9.93 months vs. 14.4 months, p < 0.001). The same results were observed in the validation group (Figs. 5f and 10.4 months vs. 12.3 months vs. 23.2 months, p = 0.026). The ROC curve analysis results for the performance of the integrated model in predicting 1-year, 2-year, and 3-year PFS probability are shown in Fig. 6e **and f**. In the training cohort, the AUC values for the prediction of 1-year, 2-year, and 3-year PFS probability with this model were 0.676 (95% CI 0.568–0.783), 0.662 (95% CI 0.511–0.813), and 0.594 (95% CI 0.415–0.772), respectively. In the validation group, the corresponding AUC values were 0.642 (95% CI 0.458–0.826), 0.709 (95% CI 0.543–0.875), and 0.756 (95% CI 0.585–0.926), respectively. The C-index for the integrated model was 0.616 (95% CI 0.542–0.690) in the training group and 0.649 (95% CI 0.553–0.745) in the validation group.

The results regarding the predictive performance of the three models for the 1-year, 2-year, and 3-year PFS probability of every patient in the training and validation groups are summarized in Table 4 and 5. The integrated model combining radiomics and genomics outperformed the radiomics or genomics models for predicting PFS in ESCC patients after dCRT.

**Table 5:** Predictive performance of the three models in the validation group for PFS.

Model	AUC (95%CI)			C-index (95%CI)
	1-year PFS	2-years PFS	3-years PFS	
Rad-score	0.618 (0.446–0.789)	0.662 (0.474–0.849)	0.731 (0.529–0.933)	0.625 (0.535–0.715)
HRR pathway status	0.582 (0.440–0.723)	0.642 (0.544–0.740)	0.621 (0.535–0.708)	0.586 (0.501–0.671)
Rad_HRR	0.642 (0.458–0.826)	0.709 (0.543–0.875)	0.756 (0.585–0.926)	0.649 (0.553–0.745)

Abbreviations: PFS progression-free survival, AUC area under curve

## Discussion

In this study, we developed three models for predicting the efficacy of dCRT in ESCC patients, including a CT-based radiomics model, a genomics model, and an integrated model combining both radiomics and genomics futures. We then confirmed and compared the predictive performance of these models in a validation cohort. Our results demonstrated that stratification of patients into high and low progression risk groups could be achieved based on CT-based radiomic features and HRR pathway mutation status, and these features had significant value for predicting PFS among ESCC patients. Importantly, the integrated model combining the radiomics features and genomics signature was superior to either model based on only one type of data.

Radiomics is an emerging image analysis method that can extract a great number of quantitative features from imaging data to quantify tumor heterogeneity, which is significant for personalized oncology [13–15]. Several radiomics studies have investigated the role of CT-based radiomics features in predicting treatment response [16, 17] and prognosis in EC patients [7, 18, 19]. Ganeshan et al. [20] first researched the CT radiological features of patients with EC before treatment and suggested that radiological signatures representing homogeneity parameters differed significantly between stage III/IV and I/II disease and could serve an independent predictors of prognosis. Larue et al. [7] developed a random forest model predicting 3-year overall survival (OS) based on pre-treatment CT radiomic signatures and validated it in two independent cohorts of ESCC patients with AUC values ranging from 0.61 to 0.69. Hou et al. [21] retrospectively explored pre-treatment CT images of 49 ESCC patients undergoing CRT and reported that CT data could predict the response of tumors to CRT with an AUC ranging from 0.69 to 0.73. All of these studies demonstrated that radiomic features can be useful for assessing the prognosis and treatment response of EC, a finding also supported by our results. However, these previous studies included only clinical and imaging factors, not considering molecular signatures, and their findings may be further limited by their small sample sizes and lack of independent validation cohorts. Additionally, the effectiveness of radiomics for predicting progression risk in ESCC patients treated with dCRT had not been determined. In the present study, our CT-based radiomics model was found to predict PFS in ESCC patients after dCRT with a C-index of 0.587), and its performance was confirmed in a validation group (C-index = 0.625). Thus, our study supports current evidence for the value of radiomics in predicting progression of locally advanced ESSC after dCRT.

Abnormalities in genes regulating cell cycle progression have been implicated in the development of a variety of cancers, including ESCC [22, 23]. Moreover, some genetic alterations are known to affect the efficacy of treatment for cancer patients. Research revealed that the HRR pathway is associated with the sensitivity of patients with different cancer types to chemotherapy [24]. A previous study in a large pan-cancer cohort of the Cancer Genome Atlas (TCGA) showed that mutations linked to the HRR pathway are associated with inferior clinical outcomes [25]. Similarly, we previously investigated the relationship between genetic characteristics and disease outcome in EC patients treated with dCRT and found that HRR pathway alterations can be used as a prognostic marker for PFS [11]. Therefore, the introduction of genetic features is expected to further improve the reliability and accuracy of a radiomics prediction model developed on a limited training data set. A study conducted by Xie CY et al. [26] proposed a genomics-based feature selection approach to create CT-based radiomics model using differentially expressed genes to reduce the number of radiomic features. The results showed that the radiomic signature with differentially expressed genes feature selection achieved better performance for disease-free survival prediction than without. Through Cox univariate analysis in patients with primary colorectal cancers, Badic et al. [27] found that ABCC2 mRNA level, stage III, node status (N), and radiomic features, including flatness, sum entropy (SENTR), entropy from grey-level-co-occurrence-matrix (Entropy_GLCME_), and grey-level non-uniformity (GLNU_L_) are predictive factors for PFS. Further multivariate analysis identified Entropy_GLCME_, ABCC2, and Stage III as independent prognostic factors for PFS in this population (p = 0.0001). From the Cox model, the combination of clinical and radiologic features in their study was associated with a hazard ratio (HR) greater than 22, while lower HR values were observed for the different types of features individually. To the best of our knowledge, our study is the first to explore the predictive value of radiogenomics for EC survival. Multivariate Cox regression analysis demonstrated that the Rad-score developed in our study and the HRR pathway alterations identified in our study were independent prognostic factors for PFS in ESCC patients treated with dCRT. Our integrated model incorporating the radiomic signature and HRR pathway mutation status offered even more significant predictive prognostic performance than the radiomics model (C-index, 0.616 vs. 0.587) and the genomics model (C-index, 0.616 vs. 0.557) in the training cohort. Therefore, integration of radiomics models with genetic predictors has potential advantages for predicting the risk of progression in ESCC patients receiving dCRT.

Although our study established and validated prognosis prediction models, it has several limitations. First, the study was conducted on a relatively small sample size of patients, which may limit the generalizability of the results. Second, the study was conducted in a single center, which may limit the generalizability of the findings to other centers with different patient populations and imaging protocols. Third, the study design was retrospective, which may introduce bias and limit the ability to control for confounding variables. Last, the study did not include external validation. The integrated prediction model developed in this study needs to be further validated by data for a larger sample size collected from more medical centers.

## Conclusion

In summary, this study explored the utility of radiomics and genomics models as a feasible approach to predict the PFS of patients with locally advanced ESCC treated with dCRT. Compared with either the radiomics model or genomics model, the integrated model combining both types of data offered superior predictive performance. We conclude that our integrated model may be useful for early screening to identify ESCC patients at high risk of progression in order to guide more effective personalized treatment and closer follow-up to prevent progression.

## Data Availability

The datasets used and analyzed during the current study are available from the corresponding author on reasonable request.

## Abbreviations

CT: Computed tomography
HRR: Homologous recombination repair
ESCC: Esophageal squamous cell carcinoma
EC: Esophageal carcinomas
dCRT: Definitive chemoradiotherapy
LASSO: Least absolute shrinkage and selection operator
PFS: Progression-free survival
AUC: Area under curve
ROC: Receiver operating characteristic
MR: Magnetic resonance
PET: Positron emission tomography
ROI: Region of interest
ICC: Inter-class correlation coefficient
MSE: Mean square error
GLCM: Gray level co-occurrence matrix
GLDM: Gray level dependence matrix
GLRLM: Gray level run-length matrix
GLSZM: Gray level size zone matrix
NGTDM: Neighbor gray tone difference matrix
